# Parameter calibration of the simulation model for coated rice seeds in UAV seeding based on discrete element methodology

**DOI:** 10.1371/journal.pone.0361229

**Published:** 2026-11-06

**Authors:** Yu Qingxu, Mengxiao Liu, Yan Gong, Xiao Chen, Jianling Hu

**Affiliations:** 1 Nanjing Institute of agricultural mechanization, Ministry of Agriculture and Rural Areas, Nanjing, China; 2 Key Laboratory of Modern Agricultural Equipment, Ministry of Agriculture and Rural Affairs, Nanjing, China; 3 Institute of Western Agriculture, Chinese Academy of Agricultural Science, Changji, China; China University of Mining and Technology, CHINA

## Abstract

The discrete element method (DEM) is widely used in agricultural mechanization, but accurate parameter calibration for UAV seeding simulation of coated rice seeds remains challenging due to empirical parameter selection and single-stage optimization in existing studies. This study combined physical and simulation experiments to calibrate DEM parameters for coated rice seeds (CRS). Physical tests measured the intrinsic parameters and actual repose angle (18.84°). A three-stage progressive optimization strategy was then adopted: Plackett–Burman test to screen significant parameters, steepest ascent test to narrow the optimal range, and quadratic rotation orthogonal combination test to establish a regression model. Four significant parameters were identified: the CRS-CRS coefficient of restitution (C), the CRS-CRS coefficient of static friction (D), the CRS-CRS coefficient of rolling friction (E), and the CRS-stainless steel coefficient of static friction (G). The optimal combination was determined as 0.35, 0.22, 0.09, and 0.51, respectively. Verification using static repose angle, dynamic repose angle, and UAV total seeding rate showed average relative errors below 5% for all three indices. These results demonstrate that the calibrated parameters are reliable for DEM simulation of coated rice seeds, and the proposed multi-stage method effectively improves calibration accuracy and model applicability for UAV seeding operations.

## 1. Introduction

Multi-rotor UAVs offer significant advantages for agricultural operations such as plant protection and seeding. Compared with ground agricultural machinery, UAVs are less restricted by field trafficability. Compared with manned aircraft, UAVs require no dedicated airports. Therefore, UAVs can take off and land flexibly in the field, allowing timely responses to the operating environment and ensuring high operational efficiency. They are particularly suitable for hilly terrain, mountains, and small-scale plots where conventional equipment is difficult to operate, while also effective for large-scale spreading in open plains when operating in coordinated multi-UAV teams [1–4]. In recent years, with advancing multi-rotor UAV technology, granular spreading applications have expanded to seeds, fertilizers, herbicides, feed, and veterinary drugs [5–8]. Commercially mature and advanced UAV spreading systems include the DJI T100S, Jifei sixth‑generation RuiBo system, and Topcon “Fengpeng” system. As UAV seeding technology matures, specially coated rice seeds via pelleting and coating treatment have been increasingly adopted. The coating provides disease and insect resistance, weeding and bird repellent functions, while maintaining uniform particle size and surface roughness, improving particle flowability, reducing airflow resistance, and effectively optimizing UAV seeding performance.

The quality of UAV speeding operations is primarily evaluated by the coefficient of variation of uniformity. In this regard, China has established the industry standard NY/T3881-2021 for assessing UAV seeding quality [9]. However, due to the irregular shape of rice seeds and the high uniformity requirements, the “bottleneck” of seeding uniformity for UAV rice sowing remains to be addressed. Domestic and foreign scholars have carried out extensive studies on UAV seeding mechanism and structural optimization [10–14]. Restricted by the irregular shape of rice grains and high uniformity requirements, the uniformity bottleneck still restricts the popularization of UAV rice direct seeding. Most existing studies mainly focus on the structural design and prototype development of UAV rice seeding devices [15–19]. The discrete element method (DEM) is widely applied in agricultural mechanization research [20,21]. Establishing DEM simulations for UAV-based seeding of coated rice seeds facilitates mechanistic study of seeding uniformity, but requires first developing a discrete element model of the coated rice seeds. Numerous DEM parameter calibration studies have focused on agricultural materials such as soil, fertilizers, and seeds [22–25]. Zhengfa Chen et al. [26] adopted the multi-sphere modeling method and took the repose angle as the evaluation index to calibrate discrete element parameters of long-grain rice with different moisture contents. Zhang Rongfang et al. [27] investigated the effect of filling sphere particle radii on inter-particle static and rolling friction coefficients of rice seed models, finding that optimal radius combinations yield accurate repose angle simulations. Wang Jinwu et al. [28] established a refined rice seed DEM model via 3D scanning to investigate seed accumulation characteristics under different moisture contents. They classified the accumulation process into four stages and revealed the effects of moisture content on seed accumulation morphology and internal force distribution. Mou Jiahong et al. [29] developed a rice seed DEM model suitable for fluid–solid coupling simulations, demonstrating that the model predicts particle–fluid interactions effectively. Lu Fangyuan et al. [30] calibrated the key DEM contact parameters of rice bud seeds with varying moisture contents using friction angle tests and regression analysis. The calibrated parameters were well validated with low simulation errors for multiple rice varieties, providing an effective parameter reference for dynamic simulation of rice precision seeding devices. Shun Zhang et al. [31] established a shape approximation and contact parameter calibration method for rice seed discrete element models to predict the seeding performance of mechanical seed-metering devices. They identified inter-particle static friction and rolling friction coefficients as the dominant parameters affecting rice seed metering performance.

Few studies have focused on DEM modeling and parameter calibration for coated rice seeds, while most existing research on conventional grains, soil, and fertilizers adopts a single-sphere particle simplification. To address this gap, this study establishes a multi-sphere aggregated DEM model to characterize the actual contour of coated rice seeds, providing a theoretical basis for UAV seeding optimization. A combined physical and simulation-based approach was adopted: basic physical parameters and repose angle of coated rice seeds were measured, contact parameter ranges were determined via literature review and preliminary experiments, and key contact parameters were calibrated using Plackett–Burman, steepest ascent, and quadratic regression tests. The reliability of the calibrated parameters was verified through static and dynamic tests, ensuring applicability for UAV seeding simulation.

## 2. Materials and methods

### 2.1. Experimental materials

#### 2.1.1. Determination of basic physical characteristics of seeds.

The experimental rice seed variety is Zhongzao 39, and all coated rice seeds were self-prepared in our laboratory. Based on preliminary agronomy-mechanism integration tests, coated rice seeds with a coating thickness of 0.6 ± 0.2 mm were selected as the test samples, from which impurities, broken, and damaged seeds were removed by screening. One hundred seeds were randomly selected for triaxial dimension measurement using a digital vernier caliper (accuracy: 0.01 mm). The measured dimensions were as follows length 7.5 ± 0.5 mm, width 3.8 ± 0.2 mm, thickness 3.3 ± 0.2 mm. A 5 g test sample was randomly weighed, and its moisture content was measured using an LHS16-A drying method moisture meter (accuracy: ± 0.2%). The measurement was repeated five times, yielding an average moisture content of 6.4%. For thousand-grain weight measurement, 1000 seeds were randomly selected each time and weighed using a Mettler Toledo ML4002T electronic balance (accuracy: 0.01 g). The average value from five replicate measurements was 41.12 g. To obtain the outer contour profile, coated rice seeds with triaxial dimensions close to the average values were selected. The contour dimensions were measured and mapped using a JSD vertical microscope. The physical appearance and contour curve of the coated rice seeds are shown in Fig 1.

**Fig 1:**
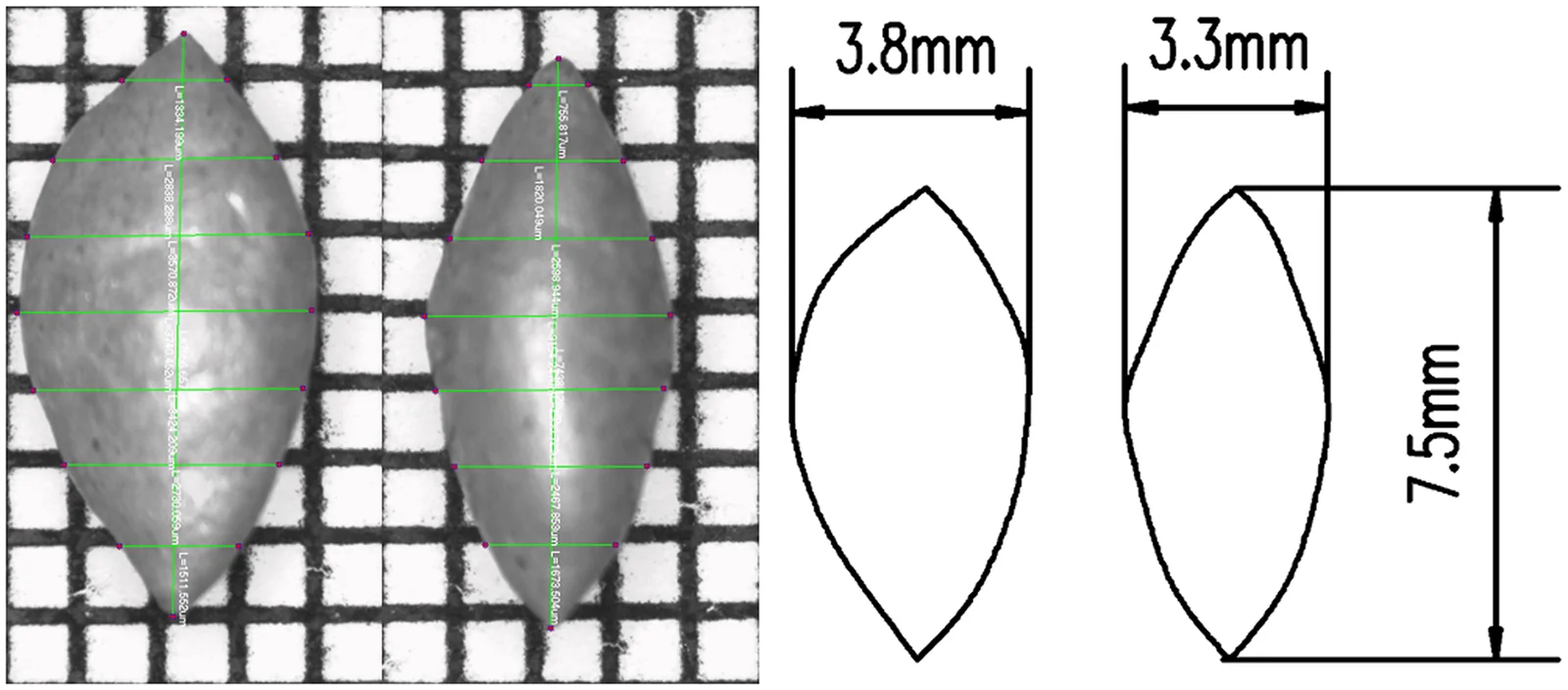
Physical appearance and contour profile of coated rice seeds.

#### 2.1.2. Establishment of the discrete element particle model for coated rice seeds.

In discrete element simulation software, spherical particles can be used to construct discrete element models of materials. A spherical particle has only one radius parameter and exhibits a single type of contact state with other spheres, making contact detection straightforward. This significantly reduces computation time and contributes to stable program operation. For materials with complex shapes, multiple spherical particles can be combined to create a discrete element model that approximates the actual shape. As the number of constituent spherical particles increases, the model becomes more geometrically accurate; however, this also substantially prolongs simulation time and reduces computational efficiency [32,33]. Based on simulation experience, it is not always necessary to perfectly replicate the exact shape of the real material. Considerations such as computational capacity and processing speed must be taken into account, and more critically, the interaction parameters between materials and between material and geometric boundaries play a decisive role. In this study, a discrete element model of coated rice seeds was constructed using a combination of 11 spherical particles, as shown in Fig 2. The 11-sphere model was constructed by manual tight filling according to the actual contour of coated rice seeds. The dimensions and positions of each spherical particle are detailed in Table 1. Since the coating layer is thin and uniformly wrapped, the overall physical properties of the coated seed are treated as a uniform particle for simulation efficiency.

**Table 1:** The position and size of each spherical particle.

NO.	coordinate position			radius/mm
	X/mm	Y/mm	Z/mm	
1	0	0.3	0	1.6
2	0	−0.3	0	1.6
3	1	0.3	0	1.5
4	1	−0.3	0	1.5
5	2	0	0	1.2
6	−2	0	0	1.2
7	3	0	0	0.75
8	−3	0	0	0.75
9	−1	0.3	0	1.5
10	−1	−0.3	0	1.5
11	0	0	0	1.65

**Fig 2:**
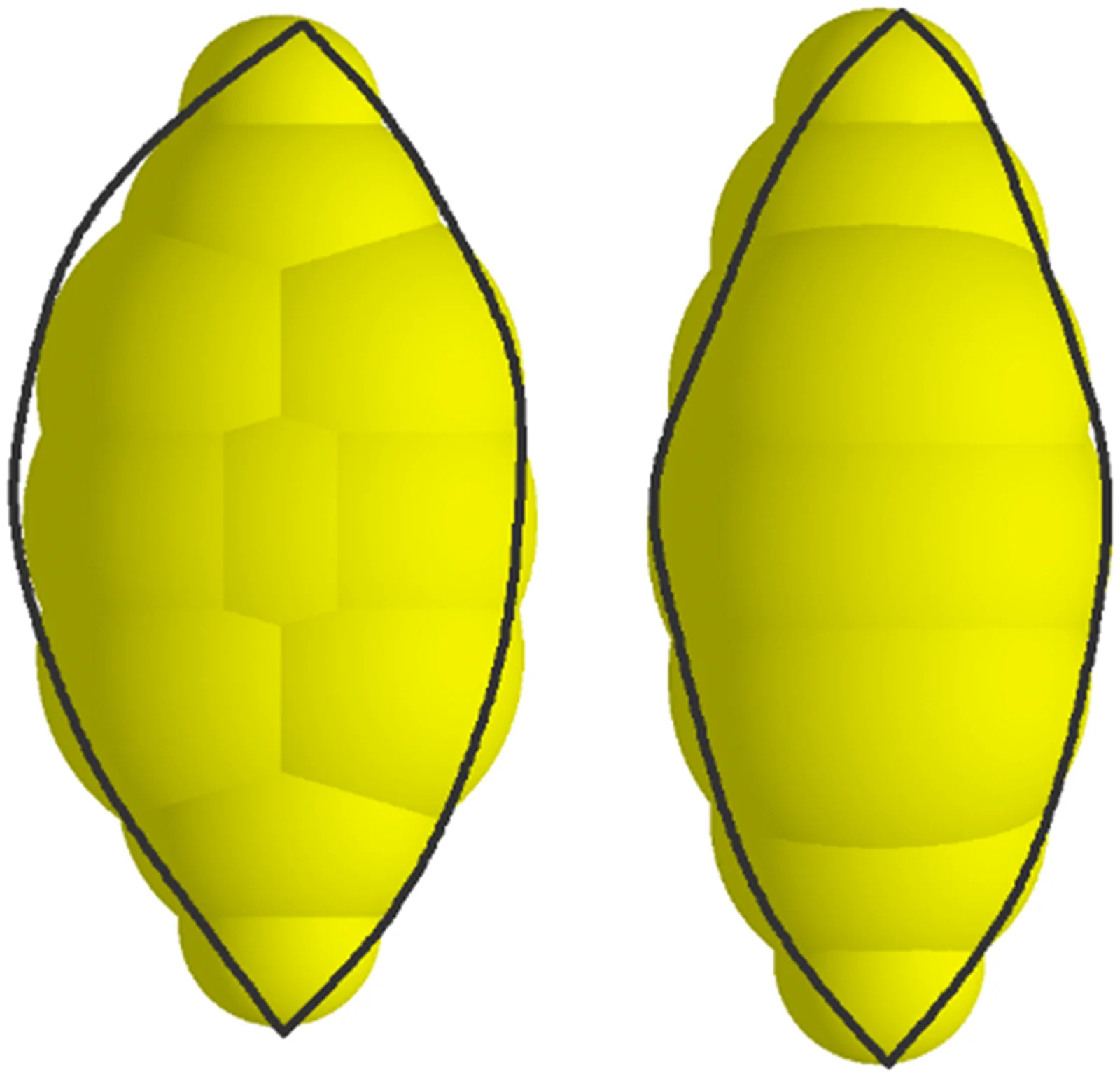
Established by stacking 11 spherical particles.

### 2.2. Physical experimentation and simulation modeling

#### 2.2.1. Repose angle test of coated rice seeds.

The repose angle is a macroscopic parameter that characterizes the flowability and frictional properties of granular materials. It is influenced by the physical properties of both the material itself and the contact surface. A smaller repose angle indicates better flowability. Common methods for measuring the repose angle include the cylinder lifting method, cylinder rolling method, draw plate method, and funnel method [34–36]. In this study, the funnel method was adopted to measure the repose angle of coated rice seeds. Specifically, the coated rice seeds were allowed to fall slowly from a fixed height onto a horizontal surface, forming a conical pile. The repose angle is defined as the angle between the generatrix of the cone and the horizontal plane. The test apparatus mainly consisted of a stainless steel funnel, a baffle plate, and a base plate. The funnel had an upper opening diameter of 250 mm, a lower opening inner diameter of 50 mm, and a cone angle of 70°. The distance from the lower end of the funnel to the upper surface of the base plate was set to 50 mm, as illustrated in Fig 3. The measurement procedure was as follows: First, the lower opening of the funnel was blocked with the baffle plate. A total of 332.87 g of coated rice seeds (8,000 seeds) were poured into the funnel. The baffle plate was then quickly removed, allowing the seeds to fall freely under gravity and accumulate on the base plate, forming a seed pile. After the seeds had completely settled and remained stationary, a high-definition camera was used to capture a front-view image of the pile, as shown in Fig 4.

**Fig 3:**
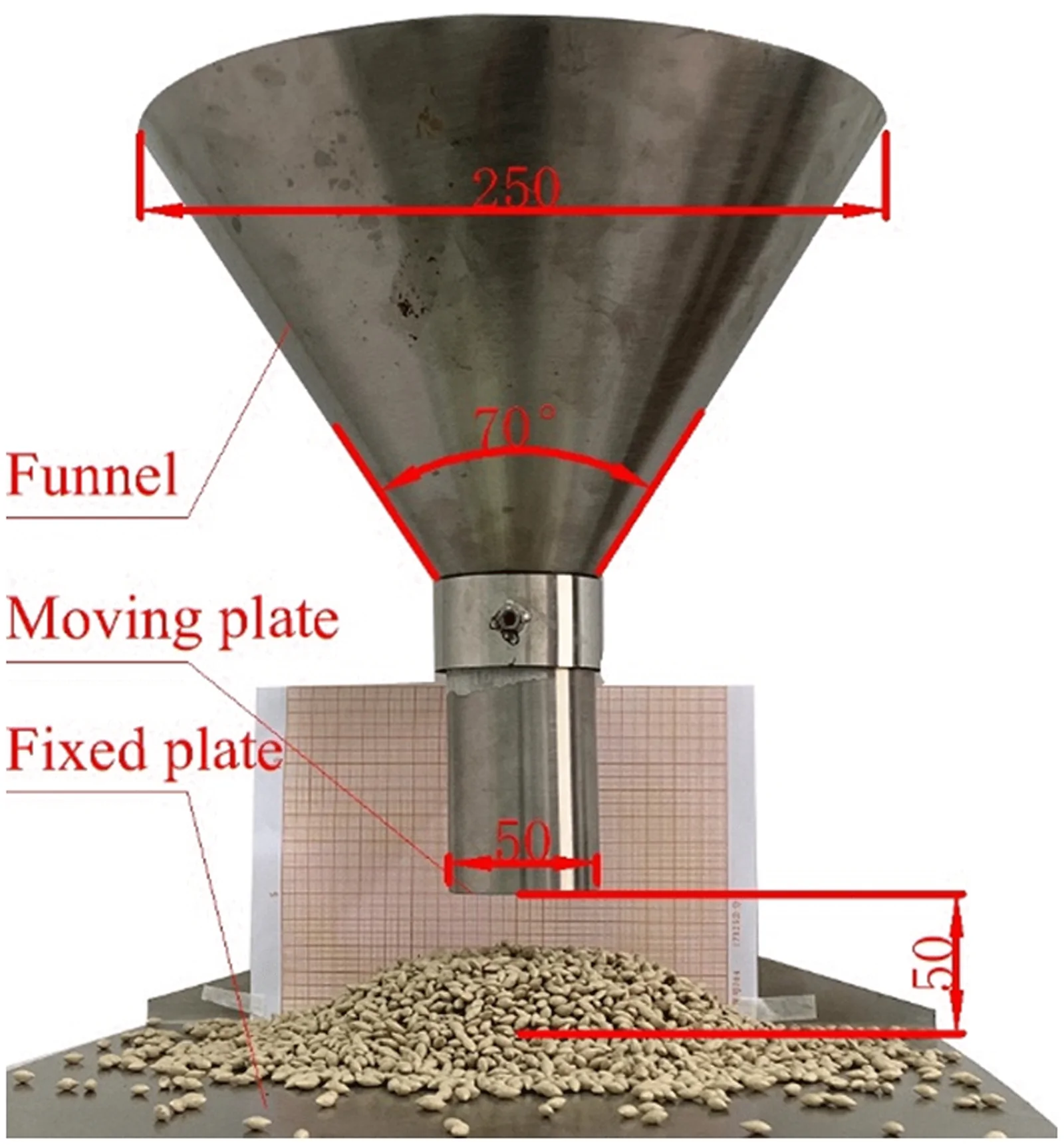
The bench test device.

**Fig 4:**
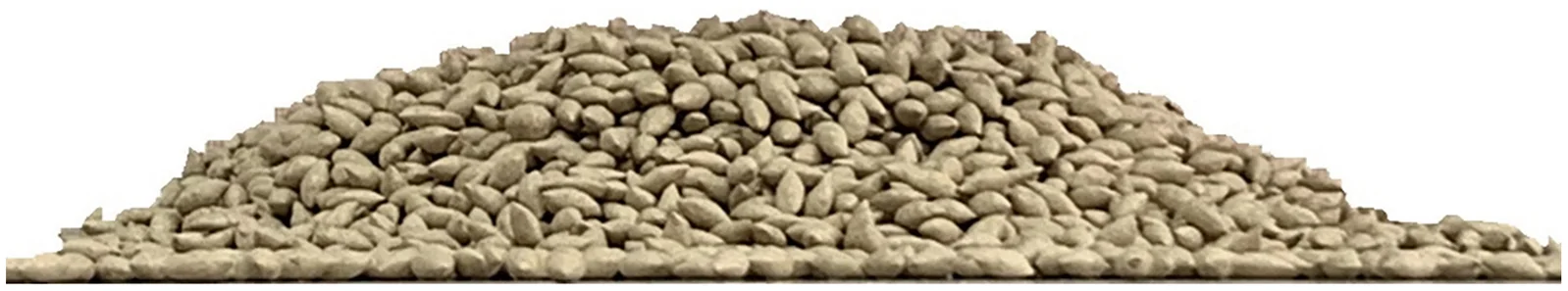
Coated rice seed stack.

To minimize measurement errors caused by human factors, computer image processing technology was employed to analyze the captured images. MATLAB software was used to process the left and right side images of the coated rice seed pile. Each image underwent denoising, grayscale conversion, and binarization. Edge detection was then applied to extract the boundary pixels, and the connection of these pixels formed the contour curve of the seed pile. Finally, the least squares method was used to perform linear fitting on the contour curve, where the slope of the fitted line corresponds to the tangent of the repose angle. The repose angle was calculated as the average of the left and right side measurements. The complete measurement process is illustrated in Fig 5. The measured repose angle of the coated rice seeds obtained by the funnel method was determined to be 18.84°.

**Fig 5:**
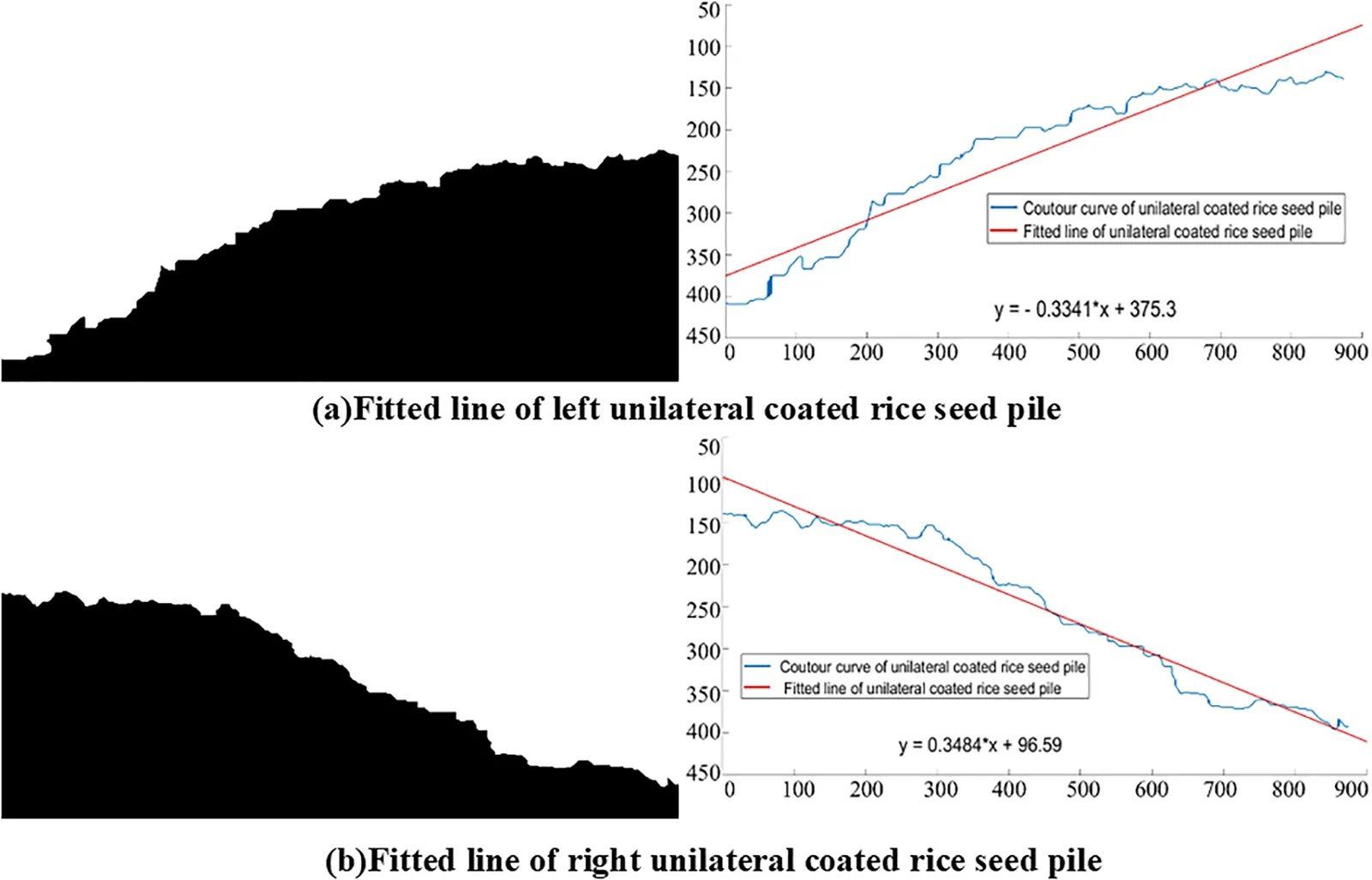
Repose angle measurement.

#### 2.2.2. Discrete element simulation modeling and Simulation setup.

The solid models of the funnel, baffle plate, and bottom plate were established in 3D design software and subsequently imported into the discrete element simulation environment. The material of the funnel, baffle plate, and bottom plate was set to stainless steel. The total simulation time was configured as 5 s, with a mesh size of 3 Rmin, and the gravitational acceleration was adopted as 9.81 m/s^2^. The calculation time step was set to 20% of the Rayleigh time step. To balance simulation efficiency and result reliability, the particle size was fixed, and a total of 8,000 coated rice seed particles were generated. After all particles were generated and the average velocity of the entire particle group approached 0 m/s—indicating a stable state—the simulation proceeded. Fig 6 shows the average velocity curve of all particles during the 2nd second. At the 3rd second, the baffle plate began translational movement along the X-axis at a speed of 3 m/s, thereby removing the baffle from the funnel outlet. The coated rice seed particles then fell freely under gravity. The complete simulation process of the repose angle test is illustrated in Fig 7, culminating in the formation of a seed pile on the bottom plate. Upon completion of the simulation, the same computer image processing technique was applied to analyze the captured images and determine the simulated repose angles on both the left and right sides of the seed pile.

**Fig 6:**
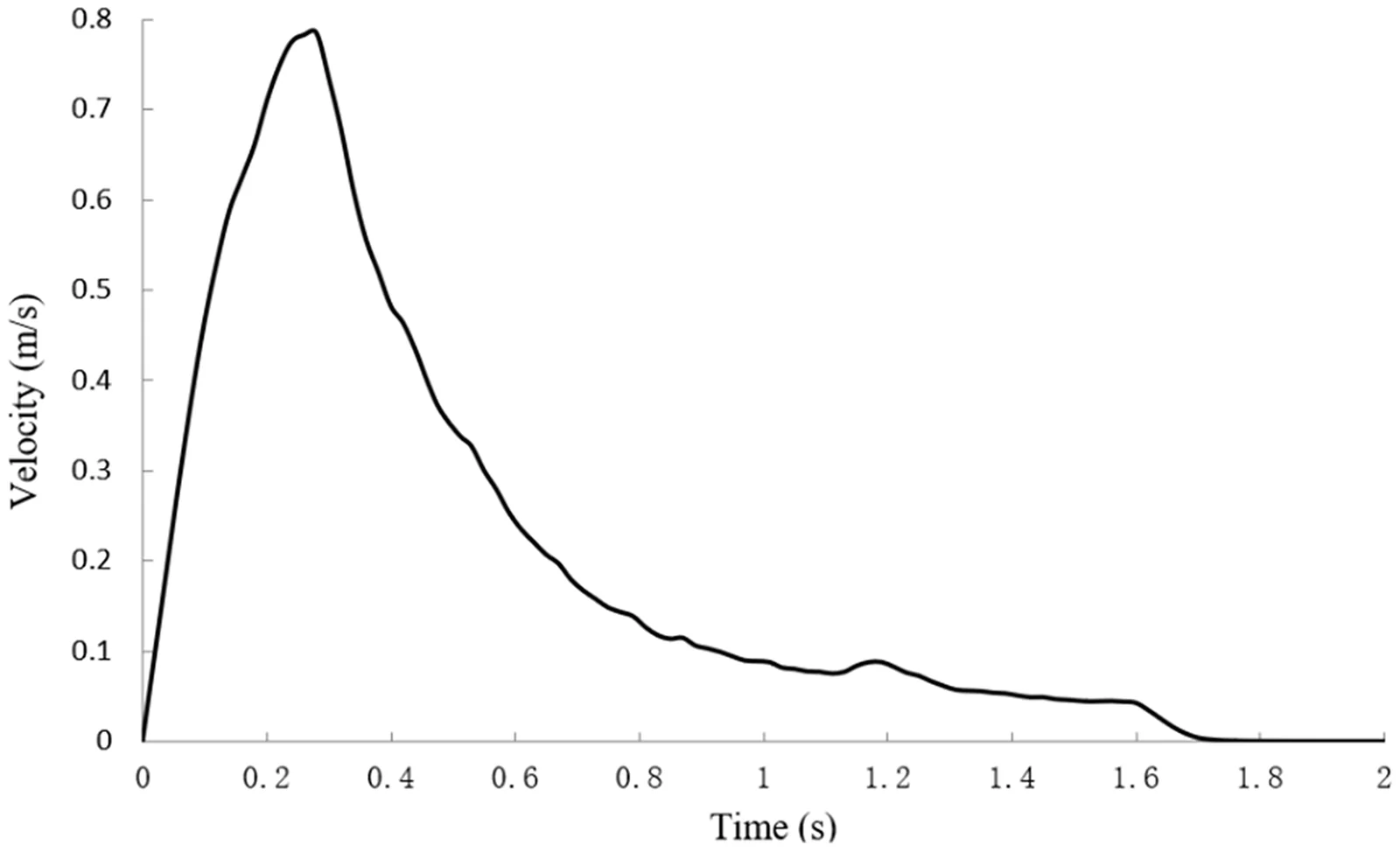
Average velocity curve of all particles within 0 to 2 seconds.

**Fig 7:**
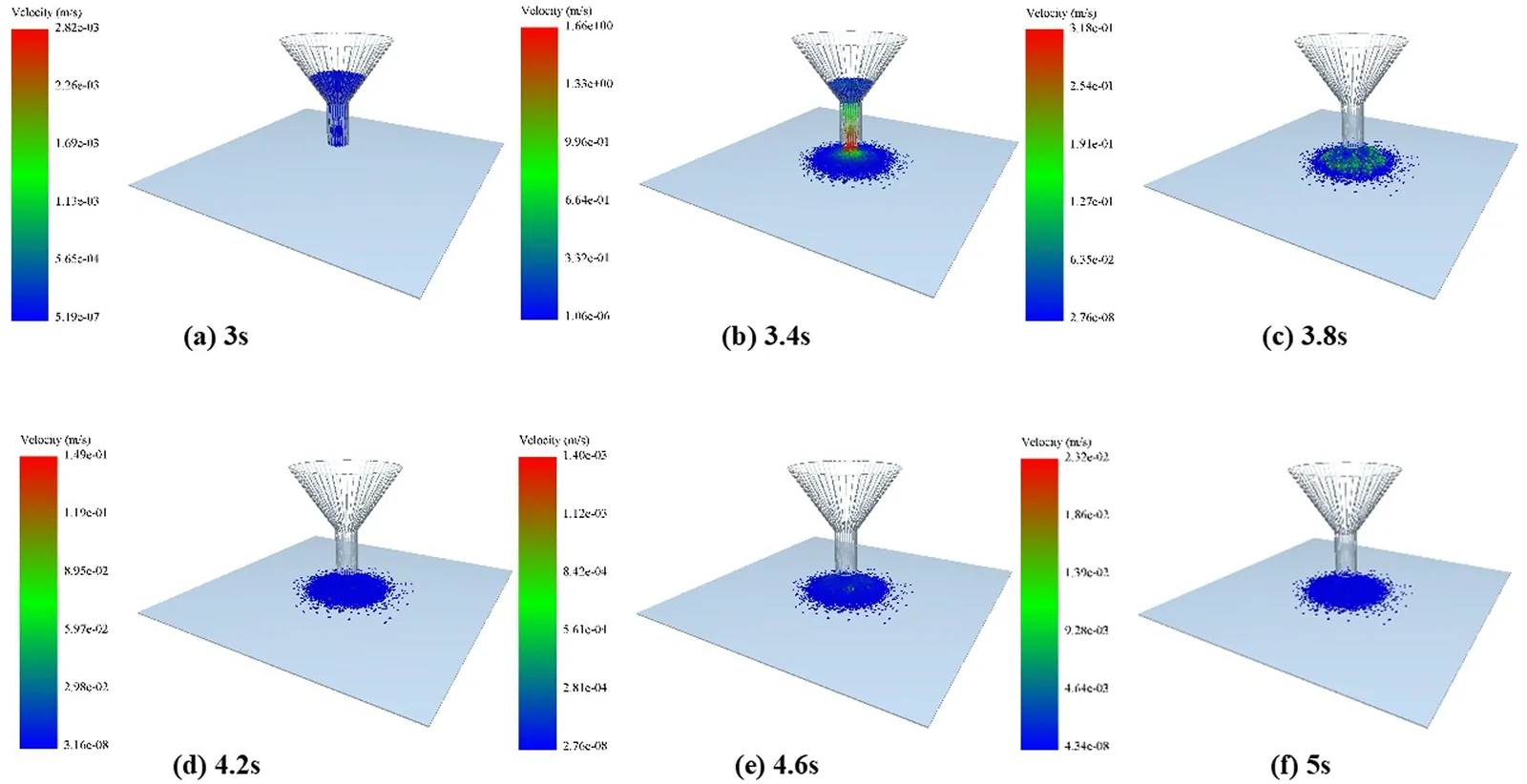
Simulation test process of rice seed accumulation angle during coating within 3 to 5 seconds.

Prior to the simulation tests, the contact model for the particles was selected, and the relevant simulation parameters for the coated rice seeds and the stainless steel contact material were configured. The simulation parameters are listed in Table 2. The seed particle density was calibrated as 965 kg/m^3^ for discrete element simulation, rather than adopting the true physical density of coated rice seeds. This calibrated density was set to ensure that the thousand-grain weight of the established particle model is in good agreement with the measured thousand-grain weight of actual coated rice seeds. Based on relevant literature [26–30,37], the Poisson’s ratio of stainless steel was taken as 0.3, the density as 7.850 × 10^3^ kg/m^3^, and the shear modulus as 7.9 × 10^10^ Pa. The ranges of the remaining simulation parameters were also determined from the literature.

**Table 2:** Simulation parameters.

Parameter	Numerical value	Remark
Poisson’s ratio of coated rice seed	0.2 ~ 0.4	Awaiting calibration
Density of coated rice seed(kg/m^3^)	965	Known
Shear modulus of coated rice seed(pa)	1.81E + 7 ~ 1.81E + 8	Awaiting calibration
Poisson’s ratio of stainless steel	0.3	Known
Density of stainless steel(kg/m^3^)	7850	Known
Shear modulus of stainless steel(pa)	7.9E + 10	Known
CRS-CRS coefficient of restitution	0.1 ~ 0.5	Awaiting calibration
CRS-CRS coefficient of static friction	0.1 ~ 0.3	Awaiting calibration
CRS-CRS coefficient of rolling friction	0.05 ~ 0.15	Awaiting calibration
CRS-stainless steel coefficient of restitution	0.2 ~ 0.6	Awaiting calibration
CRS-stainless steel coefficient of static friction	0.3 ~ 0.7	Awaiting calibration
CRS-stainless steel coefficient of rolling friction	0.1 ~ 0.15	Awaiting calibration

#### 2.2.3. Selection of contact parameters.

The appropriate contact model should be selected based on the characteristics of the simulated granular materials. Commonly used contact models include Hertz–Mindlin (no slip), Hertz–Mindlin with JKR, Hertz–Mindlin with RVD rolling friction, and Hertz–Mindlin with bonding, each with distinct applicable scopes [38,39]. Given that coated rice seeds exhibit low moisture content and negligible adhesion between particles, the Hertz–Mindlin non‑sliding contact mechanics model was adopted in this study. During the simulation, parameters such as particle displacement, force, and velocity are determined by the minute elastic deformations occurring at contacts between particles or between particles and the contact material. In accordance with Newton’s second law, each coated rice seed particle model undergoes translational and rotational motion under the influence of net forces and torques. This model provides an accurate representation of the physical scenario, offering precise and efficient force calculations, thereby reducing simulation time and improving computational efficiency. The Hertz-Mindlin contact model is illustrated in Fig 8.

**Fig 8:**
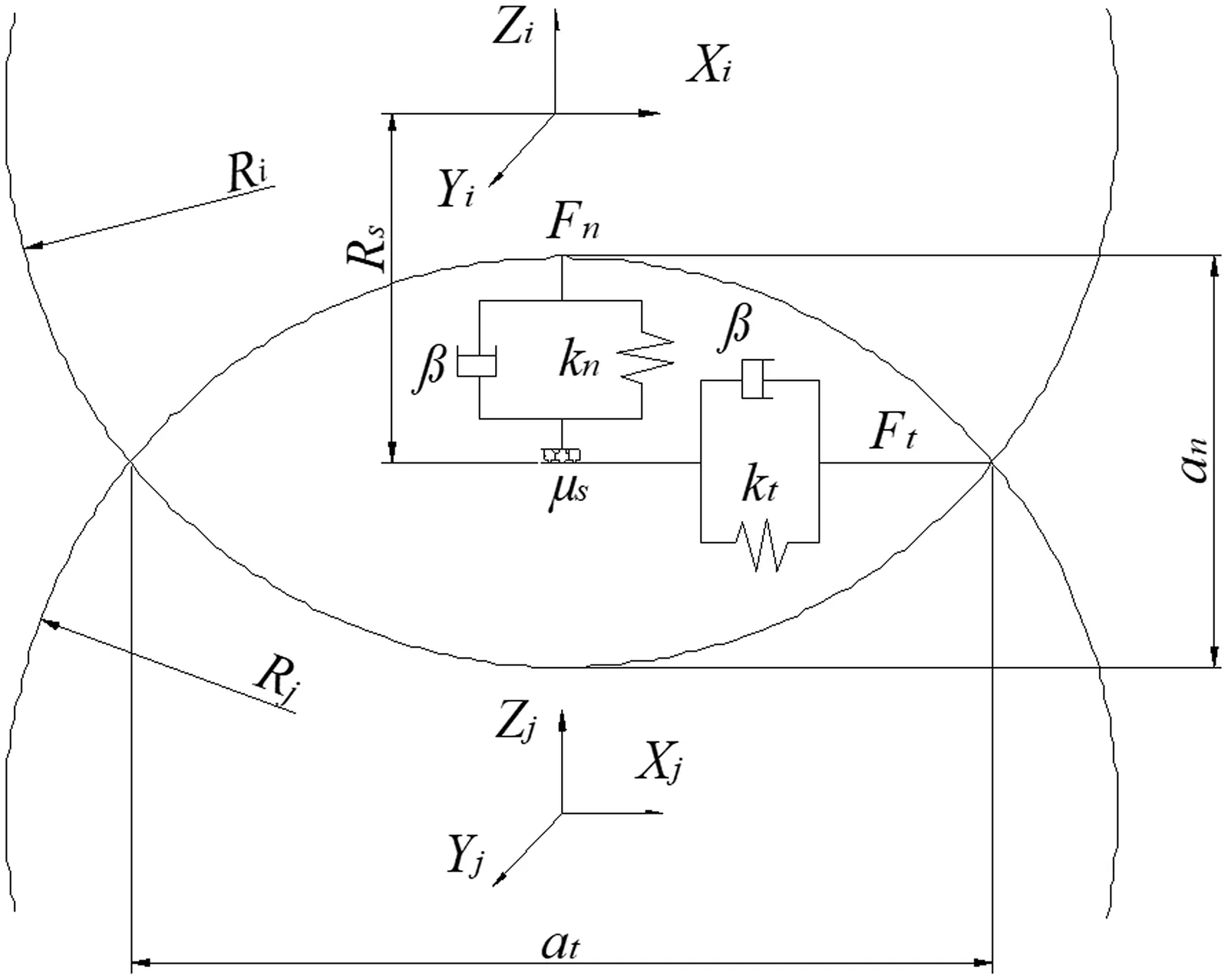
Contact model of Hertz-Mindlin.

Based on the above model, the coated rice seed particles are primarily subjected to gravity, normal force, tangential force, normal damping force, tangential damping force, Coulomb friction force, tangential moment, and rolling friction moment during the accumulation process. Under the combined action of these forces and moments, the particles undergo translational movement and rolling. When the tangential force exceeds the Coulomb friction force, sliding occurs at the contact surfaces. The rolling motion of the particles is governed by the combined effect of the tangential moment and the rolling friction moment [40].

### 2.3. Experimental design and methods

In this study, the Plackett-Burman test was first employed to screen out the simulation parameters with significant effects. Subsequently, the steepest climbing test was used to determine the level range of the significant factors. Finally, the quadratic rotation orthogonal combination test was adopted to confirm the optimal parameter combination for the discrete element simulation model of coated rice seeds.

#### 2.3.1. Plackett-Burman design experiment.

The Plackett–Burman test was designed using Design-Expert software, with the repose angle of coated rice seeds taken as the response value to identify the simulation parameters having significant effects. In the simulation experiment, a total of eight real parameters (A to H) and three dummy parameters (I to K) were considered. Each parameter was set at two levels, low and high, based on the recommended value ranges, represented by codes −1 and +1, respectively. The Plackett–Burman test parameters are listed in Table 3. One center point was included in the simulation test, and a total of 12 experimental runs were conducted. Each run was repeated three times, and the average value was taken as the repose angle for that run. The Plackett–Burman experimental design and corresponding results are presented in Table 4.

**Table 3:** Plackett-Burman test parameter table.

Symbol	Simulation parameter	Low level(−1)	High level(+1)
A	Poisson ratio of coated rice seeds	0.2	0.4
B	Shear modulus of coated rice seeds (pa)	1.81 E + 7	1.81 E + 8
C	CRS-CRS coefficient of restitution	0.1	0.5
D	CRS-CRS coefficient of static friction	0.1	0.3
E	CRS-CRS coefficient of rolling friction	0.05	0.15
F	CRS-stainless steel coefficient of restitution	0.2	0.6
G	CRS-stainless steel coefficient of static friction	0.3	0.7
H	CRS-stainless steel coefficient of rolling friction	0.1	0.15
I、J、K	Virtual parameter	−1	1

**Table 4:** Plackett-Burman test scheme and results.

Serial number	A	B	C	D	E	F	G	H	I	J	K	Repose angle θ(°)
1	−1	1	1	1	−1	−1	−1	1	−1	1	1	16.12
2	−1	−1	1	−1	1	1	−1	1	1	1	−1	7.78
3	−1	−1	−1	−1	−1	−1	−1	−1	−1	−1	−1	3.47
4	1	1	−1	1	1	1	−1	−1	−1	1	−1	23.41
5	1	1	1	−1	−1	−1	1	−1	1	1	−1	10.31
6	−1	−1	−1	1	−1	1	1	−1	1	1	1	23.29
7	1	−1	1	1	−1	1	1	1	−1	−1	−1	23.09
8	−1	1	−1	1	1	−1	1	1	1	−1	−1	30.97
9	1	1	−1	−1	−1	1	−1	1	1	−1	1	4.44
10	1	−1	−1	−1	1	−1	1	1	−1	1	1	17.35
11	−1	1	1	−1	1	1	1	−1	−1	−1	1	16.18
12	1	−1	1	1	1	−1	−1	−1	1	−1	1	22.47

Note: Variable A-K has the same meaning as Table 3, the same as below.

Plackett–Burman design was adopted in this study to rapidly screen key parameters affecting the repose angle, which only requires approximate linearity within the designed factor intervals. Non-significant factors were subsequently fixed at the midpoint of their respective value ranges for subsequent optimization. Variance analysis of the experimental results was performed using Design-Expert software to determine the significance level of each parameter, as summarized in Table 5. The results show that the CRS-CRS coefficient of restitution (C) exerted a significant effect on the simulated repose angle. Moreover, the CRS-CRS coefficient of static friction (D) and the CRS-CRS coefficient of rolling friction (E), as well as the CRS-stainless steel coefficient of static friction (G), all exhibited highly significant effects. The remaining simulation parameters showed no significant influence on the repose angle.

**Table 5:** Analysis of variance of Plackett-Burman test results.

Parameter	Degree of freedom	The adjusted sum of squares of deviation	F-value	P-value	Significance
Model	8	812.25	573.66	0.0001	**
A	1	0.89	5.00	0.1113	
B	1	1.32	7.46	0.0719	
C	1	4.06	22.94	0.0173	*
D	1	530.94	2999.83	< 0.0001	**
E	1	116.81	660.00	0.0001	**
F	1	0.52	2.94	0.1848	
G	1	157.69	890.95	< 0.0001	**
H	1	0.03	0.18	0.6992	

Note: ** indicates highly significant (P < 0.01); * indicates significant (P < 0.05). The same below.

#### 2.3.2. Steepest climbing test.

Based on the four significant parameters (C, D, E, and G) identified by the Plackett–Burman test, the relative error of the repose angle between the simulation and physical tests was used as the evaluation index. The steepest climbing test was then conducted to rapidly approach the optimal parameter region. the relative error of repose angle (*δ*) is calculated as follows:


$$\begin{array}{c} \delta =\frac{|\theta \text{-}\theta_{\text{1}}|}{\theta_{\text{1}}}\times \text{100\%} \end{array}$$ (1)

Where:

*θ*_1_— Measured repose angle, (°)

*θ*— Simulated repose angle, (°)

In the steepest climbing test, the non-significant parameters were set to their intermediate levels from the Plackett–Burman test. Specifically, the Poisson’s ratio of coated rice seeds (A) was taken as 0.3, the shear modulus of coated rice seeds (B) as 9.955 × 10^7^ Pa, the CRS-stainless steel coefficient of restitution (F) as 0.4, and the CRS-stainless steel coefficient of rolling friction (H) as 0.125. The four significant parameters (C, D, E, G) were gradually increased within their respective value ranges according to predefined step sizes. A total of five groups of steepest climbing simulation tests were conducted to determine the repose angle. Each group was repeated three times, and the average value was taken. The experimental design and corresponding results are presented in Table 6. As the values of the significant parameters increased progressively, the relative error between the simulated repose angle and the measured repose angle from the bench test first decreased and then increased. The minimum relative error was observed under the parameter combination corresponding to Test No. 3, indicating that the optimal parameter level range is located near this test.

**Table 6:** Design and results of the steepest climbing test.

No.	C	D	E	G	the repose angle θ (°)	the relative error of repose angle (*δ*) (%)
1	0.1	0.1	0.05	0.3	5.12	72.82
2	0.2	0.15	0.075	0.4	15.07	20.03
3	0.3	0.2	0.1	0.5	17.67	6.23
4	0.4	0.25	0.125	0.6	22.82	21.12
5	0.5	0.3	0.15	0.7	29.16	54.80

Note: Variable A-K has the same meaning as Table 3.

#### 2.3.3. Quadratic rotation orthogonal combination test design.

Taking the simulated repose angle and its relative error as response values, the optimal parameter combination for the coated rice seed simulation model was determined through a quadratic rotation orthogonal combination test. The coding table for the levels of significant parameters is presented in Table 7. The quadratic rotation orthogonal combination test was designed using Design-Expert software, with the non‑significant parameters set to the same values as in the steepest climbing test. A total of 27 experimental runs were conducted, and each run was repeated three times, with the average value taken. The experimental design and corresponding results for the significant parameters are shown in Table 8.

**Table 7:** Significance parameter level coding.

Coding	C	D	E	G
−2	0.2	0.15	0.075	0.4
−1	0.25	0.175	0.0875	0.45
0	0.3	0.2	0.1	0.5
1	0.35	0.225	0.1125	0.55
2	0.4	0.25	0.125	0.6

**Table 8:** Quadratic rotation orthogonal combination test scheme and results of significant parameters.

No.	C	D	E	G	θ (°)	*δ* (%)
1	0.2	0.2	0.1	0.5	18.45	2.05
2	0.35	0.225	0.0875	0.55	19.07	1.21
3	0.35	0.175	0.0875	0.45	14.63	22.33
4	0.25	0.175	0.1125	0.45	16.93	10.12
5	0.3	0.2	0.1	0.4	17.34	7.96
6	0.25	0.225	0.1125	0.45	20.22	7.32
7	0.25	0.175	0.0875	0.55	17.45	7.39
8	0.3	0.2	0.075	0.5	17.05	9.48
9	0.25	0.225	0.1125	0.55	20.57	9.18
10	0.3	0.2	0.1	0.6	19.69	4.50
11	0.3	0.2	0.1	0.5	17.64	6.35
12	0.35	0.225	0.0875	0.45	18.62	1.17
13	0.4	0.2	0.1	0.5	18.15	3.65
14	0.35	0.225	0.1125	0.45	19.27	2.31
15	0.3	0.2	0.1	0.5	17.69	6.09
16	0.25	0.175	0.1125	0.55	17.26	8.40
17	0.25	0.175	0.0875	0.45	15.96	15.31
18	0.25	0.225	0.0875	0.45	17.10	9.25
19	0.35	0.175	0.1125	0.55	17.52	7.02
20	0.25	0.225	0.0875	0.55	19.89	5.57
21	0.35	0.175	0.0875	0.55	16.13	14.37
22	0.3	0.25	0.1	0.5	20.62	9.47
23	0.35	0.225	0.1125	0.55	20.33	7.92
24	0.3	0.2	0.125	0.5	19.18	1.82
25	0.35	0.175	0.1125	0.45	16.22	13.88
26	0.3	0.15	0.1	0.5	15.39	18.29
27	0.3	0.2	0.1	0.5	19.67	4.39

## 3. Results and discussion

### 3.1. Mathematical modeling and significance testing

Design-Expert software was used to perform multiple regression analysis on the experimental data in Table 8. According to the analysis of variance (ANOVA) results for the models presented in Table 9, both regression models for the repose angle(*θ*) and the relative error of repose angle (*δ*) were highly significant, with P-values of 0.0002 and 0.0006 (P < 0.01), respectively. The lack-of-fit terms have P-values of 0.9445 and 0.1479 (both > 0.05), indicating that the equations fit the experimental data well indicating no significant lack-of-fit. The coefficients of determination R^2^ = 0.9143 and 0.8985, and the adjusted R^2^_adj_ values of 0.8143 and 0.78, are within a reasonable acceptable range for granular seed repose angle models. The relatively moderate R^2^_adj_ is mainly attributed to the inherent randomness and variability of particle stacking in center-point replicates, which is inevitable in physical repose angle tests. Meanwhile, the non-significant lack-of-fit further verifies that the established models have no overfitting, and can reliably reflect the relationship between simulation parameters and repose angle for subsequent optimization and calibration. Furthermore, the adequate precision (Adeq Precision) values are 10.480 and 10.648, indicating that both models possess good accuracy.

**Table 9:** Quadratic rotation orthogonal combination test regression model analysis of variance.

Soruce of variation	θ					*δ*				
	Mean square	Degree of freedom	Quadratic sum	P-valua	Significance	Mean square	Degree of freedom	Quadratic sum	P-valua	Significance
Model	65.14	14	4.65	0.0002	**	636.81	14	45.49	0.0006	**
C	0.73	1	0.73	0.2536		0.03	1	0.03	0.9434	
D	46.57	1	46.57	< 0.0001	**	219.19	1	219.19	< 0.0001	**
E	7.85	1	7.85	0.0020	**	27.67	1	27.67	0.0528	
G	8.13	1	8.13	0.0018	**	31.63	1	31.63	0.0405	*
CD	0.43	1	0.43	0.3783		76.96	1	76.96	0.0038	**
CE	0.01	1	0.01	0.9153		1.86	1	1.86	0.5882	
CG	0.03	1	0.03	0.8236		0.33	1	0.33	0.8191	
DE	0.24	1	0.24	0.5073		54.43	1	54.43	0.0108	*
DG	0.00	1	0.00	0.9918		50.02	1	50.02	0.0136	*
EG	0.64	1	0.64	0.2854		21.18	1	21.18	0.0847	**
C2	0.07	1	0.07	0.7235		3.33	1	3.33	0.4705	
D2	0.36	1	0.36	0.4174		119.06	1	119.06	0.0008	**
E2	0.22	1	0.22	0.5207		1.98	1	1.98	0.5759	
G2	0.00	1	0.00	0.9889		4.32	1	4.32	0.4127	
Residual error	6.11	12	0.51			71.96	12	6.00		
Lack of fit	3.42	10	0.34	0.9445		69.69	10	6.97	0.1479	
Pure error	2.68	2	1.34			2.27	2	1.13		
Total	71.24	26				708.76	26			

The regression equation between the repose angle (*θ*) of coated rice seeds and the four significant parameters (C, D, E, and G) was obtained, and a second-order regression model was established for the relative error of repose angle (*δ*) in relation to the same parameters. These equations were obtained after removing all insignificant terms. The regression equations are as follows:


$$\begin{array}{c} \theta =-\text{3}.\text{4}\text{6}\text{5}\text{2}\text{8}+\text{55}.\text{71667D}++\text{4}\text{5}.\text{7}\text{6}\text{6}\text{6}\text{7}E+\text{1}\text{1}.\text{6}\text{4}\text{1}\text{6}\text{7}G \end{array}$$ (2)

$$\begin{array}{cc} \delta = & \text{4}\text{4}\text{2}.\text{8}\text{8}\text{1}\text{3}\text{3}+\text{3}\text{5}\text{1}.\text{6}\text{2}\text{5}\text{C}-\text{2357}.\text{87667D}-\text{2186}.\text{8E}-\text{4}\text{8}\text{9}.\text{9}\text{5}\text{8}\text{3}\text{3}G\\ & -\text{1754}.\text{5CD}-\text{545CE}+\text{5902DE}+\text{1414}.\text{5DG}+\text{1841EG}+\text{3664}.\text{7}{\text{D}}^{\text{2}} \end{array}$$ (3)

### 3.2. Effects of test factors on test indicators

It can be seen from Table 9 that all interaction terms have no significant effect on the simulated repose angle (*θ*). Fig 9 illustrates the effects of individual experimental factors on the simulated repose angle (*θ*). As clearly shown in Fig 9(a), the CRS-CRS coefficient of restitution (C) tends to be horizontal, indicating no significant effect on the repose angle (*θ*). In contrast, Fig 9(b), 9(c), and 9(d) clearly show that the simulated repose angle (*θ*) increases with the CRS-CRS coefficient of static friction (D), the CRS-CRS coefficient of rolling friction (E), and the CRS-stainless steel coefficient of static friction (G). These three parameters (D, E, and G) exhibit highly significant effects on the repose angle (*θ*), which is consistent with the analysis of variance results presented in Table 9. In terms of sensitivity, the influencing priority of the four factors on repose angle θ is ranked as D > G > E > C, where D plays the dominant role.

**Fig 9:**
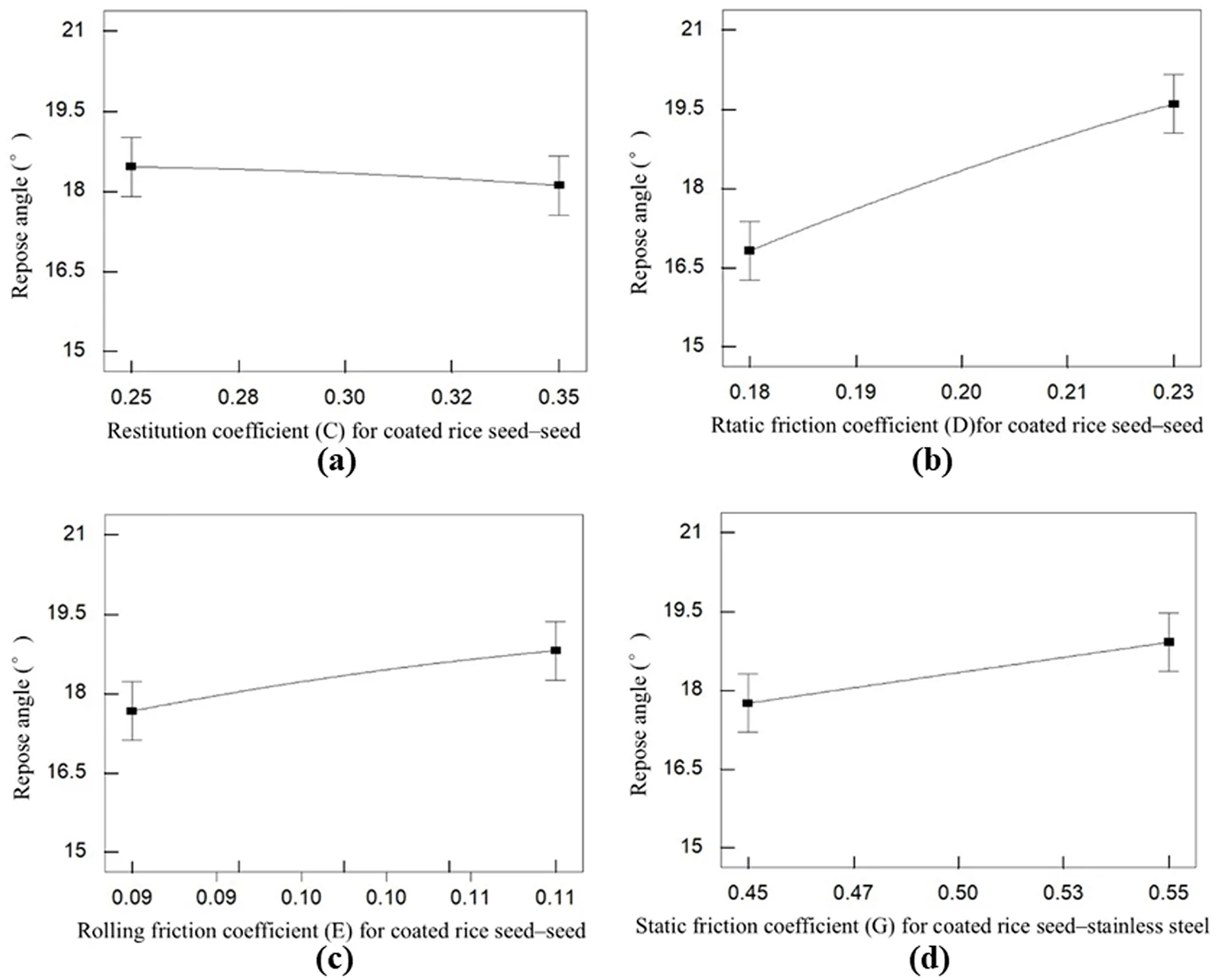
The influence of single factor on repose angle.

It can be seen from Table 9 that the interaction terms including C-D, D-E, D-G and E-G have significant effects on the repose angle error δ. Fig 10 illustrates the effects of individual experimental factors on the relative error of repose angle (*δ*). As clearly shown in Fig 10(a) and 10(c), the CRS-CRS coefficient of restitution (C) and the CRS-CRS coefficient of rolling friction (E) tend to be horizontal, indicating no significant effect on the relative error of repose angle (*δ*). In contrast, Fig 10(b) clearly shows that the simulated repose angle error (*δ*) first decreases and then increases with the CRS-CRS coefficient of static friction (D), and this parameter (D) exhibits a highly significant effect on the repose angle error (*δ*). Similarly, Fig 10(d) clearly shows that the relative error of repose angle (*δ*) first decreases and then increases with the CRS-stainless steel coefficient of static friction (G), and this parameter (G) has a significant effect on the repose angle error (*δ*). These findings are consistent with the analysis of variance results presented in Table 9. The sensitivity ranking of factors on the relative error δ follows the same order: D > G > E > C.

**Fig 10:**
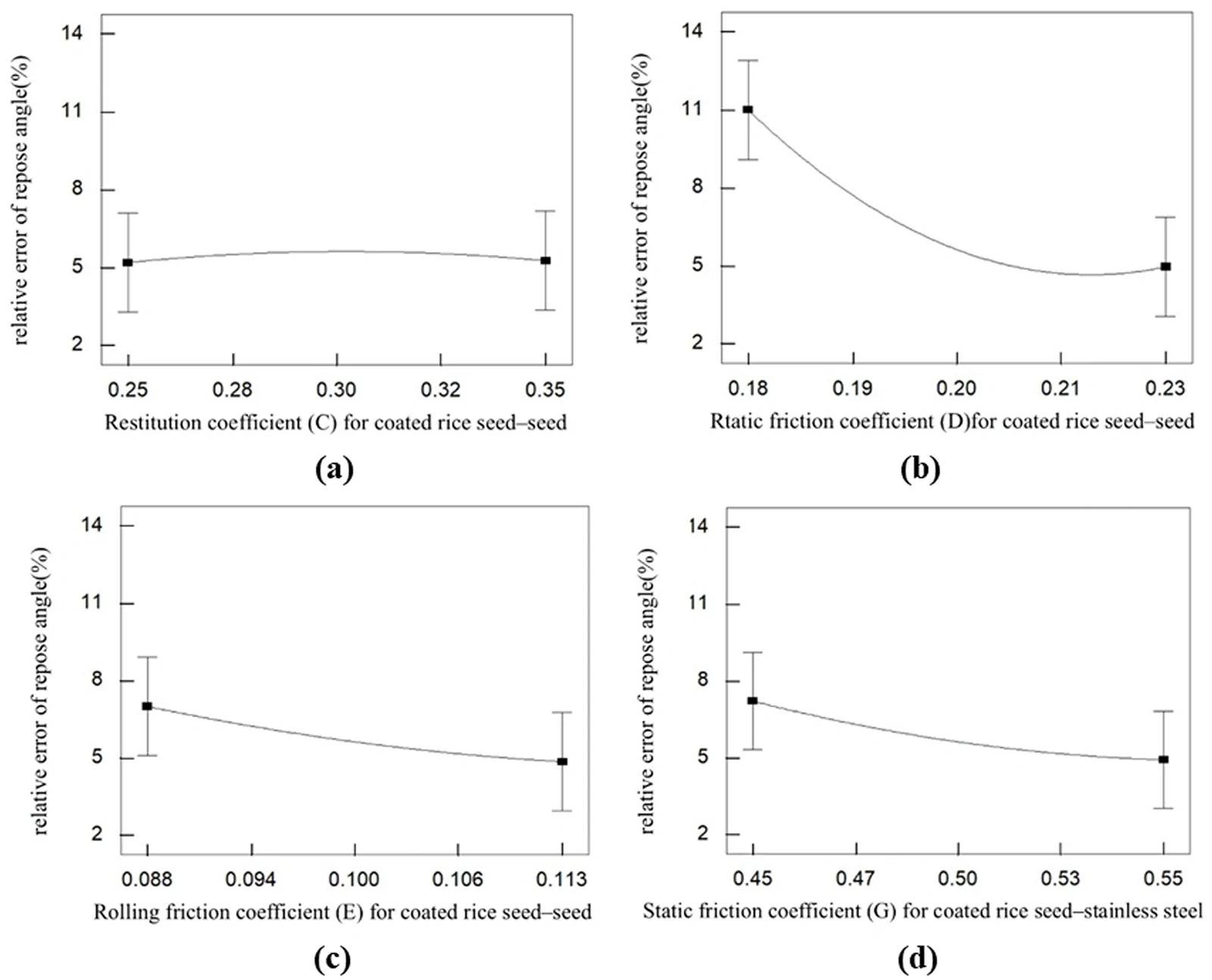
The influence of single factor on the relative error of repose angle (*δ*).

Table 9 indicates that multiple pairs of parameters produce significant interaction effects. Fig 11 presents response surface plots of interaction effects on the relative error of repose angle (δ). The surfaces of the four significant interaction terms (C-D, D-E, D-G, E-G) in Fig 11 intuitively reveal the nonlinear coupling relationships between paired factors. The morphologies of these response surfaces quantitatively explain the obvious variations in repose angle error δ caused by paired interactions, further supplementing and validating the statistical conclusions derived from the ANOVA results in Table 9.

**Fig 11:**
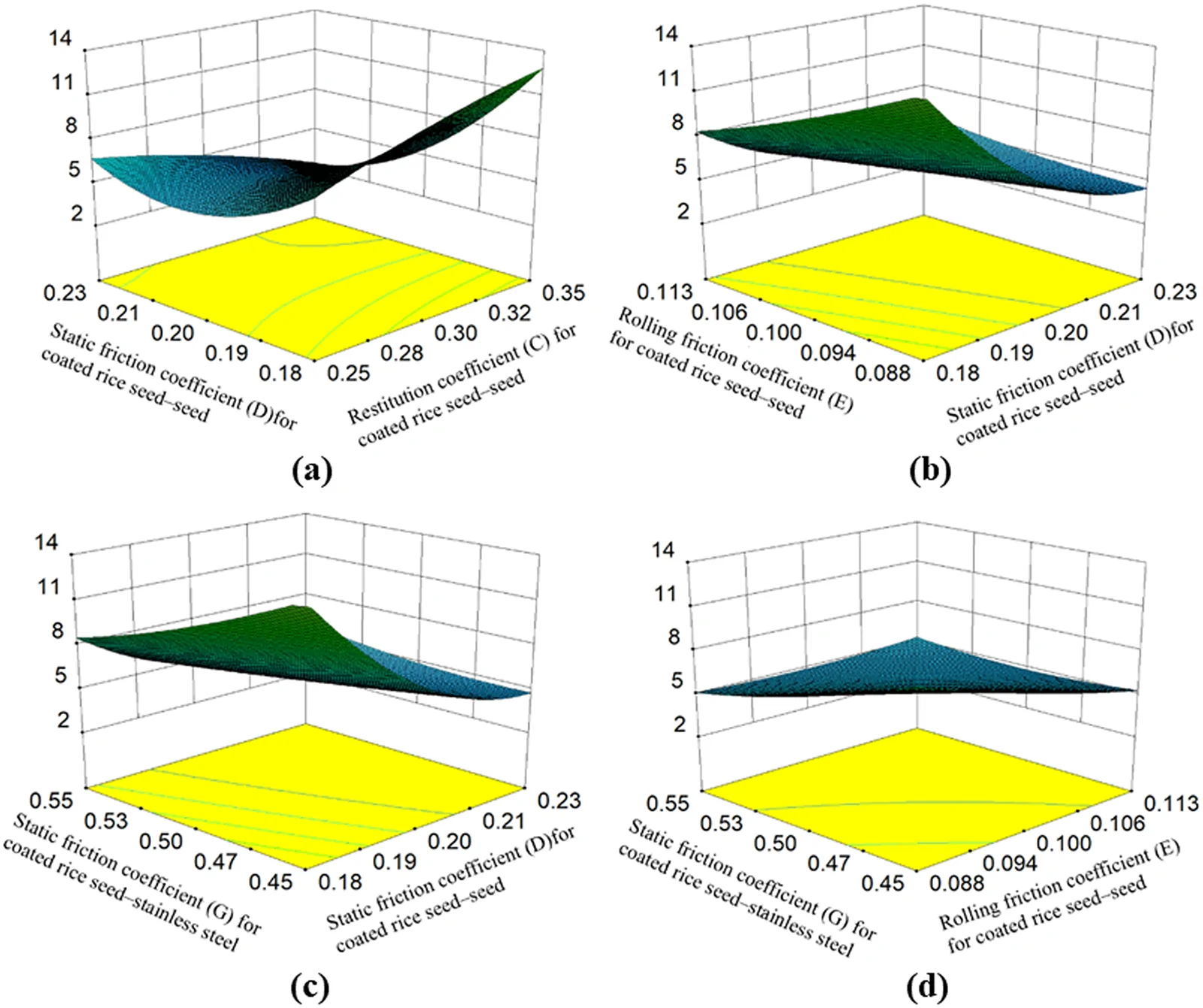
Response surface plots of interaction effects on the relative error of repose angle (δ).

### 3.3. Parameter optimization

Graphical optimization module of Design-Expert 8.0.6 software was employed to obtain feasible parameter regions. The repose angle was constrained within a reasonable interval around the physical test value (18.84°), and the relative error of the repose angle was limited to an acceptable threshold. The response surface was analyzed, and the regression model was optimized. The corresponding system of objective and constraint equations is as follows:


$$\begin{array}{c} \left\{ \begin{array}{cc} & \;\text{1}\text{8}.\text{5}{\;}^{\circ }{\leq Y}_{\text{1}}(C,D,E,G)\leq \text{1}\text{9}.\text{5}{\;}^{\circ }\\ & Y_{\text{2}}(C,D,E,G)\leq \text{5}\%\\ & s.t.\left\{ \begin{array}{cc} & \text{0}.\text{2}\leq C\leq \text{0}.\text{4}\\ & \text{0}.\text{1}\text{5}\leq D\leq \text{0}.\text{2}\text{5}\\ & \text{0}.\text{0}\text{7}\text{5}\leq E\leq \text{0}.\text{1}\text{2}\text{5}\\ & \text{0}.\text{4}\leq G\leq \text{0}.\text{6} \end{array}\right. \end{array}\right. \end{array}$$ (4)

The optimization results of the central composite design are presented in Fig 12, from which multiple sets of optimal parameters were obtained. The final selected parameter combination was as follows: coefficient of restitution for coated rice seed–coated rice seed contact: 0.35; coefficient of static friction for coated rice seed–coated rice seed contact: 0.22; coefficient of rolling friction for coated rice seed–coated rice seed contact: 0.09; and coefficient of static friction for coated rice seed–stainless steel contact: 0.51. At this optimal parameter combination, the model predicted the repose angle θ to be 18.79° (95% CI: 17.94°–19.63°), and the relative error δ to be 2.65% (95% CI: −0.25%–5.55%), indicating the reliability of the optimized solution. A simulated repose angle test was conducted using this optimal parameter combination, as shown in Fig 13. The simulation results exhibited high similarity to the measured results in terms of both repose angle and pile shape. The simulated repose angle was 18.64°, with a relative error of only 1.07%, indicating that the obtained optimal parameters are accurate and reliable, and can be used in subsequent EDEM simulation studies.

**Fig 12:**
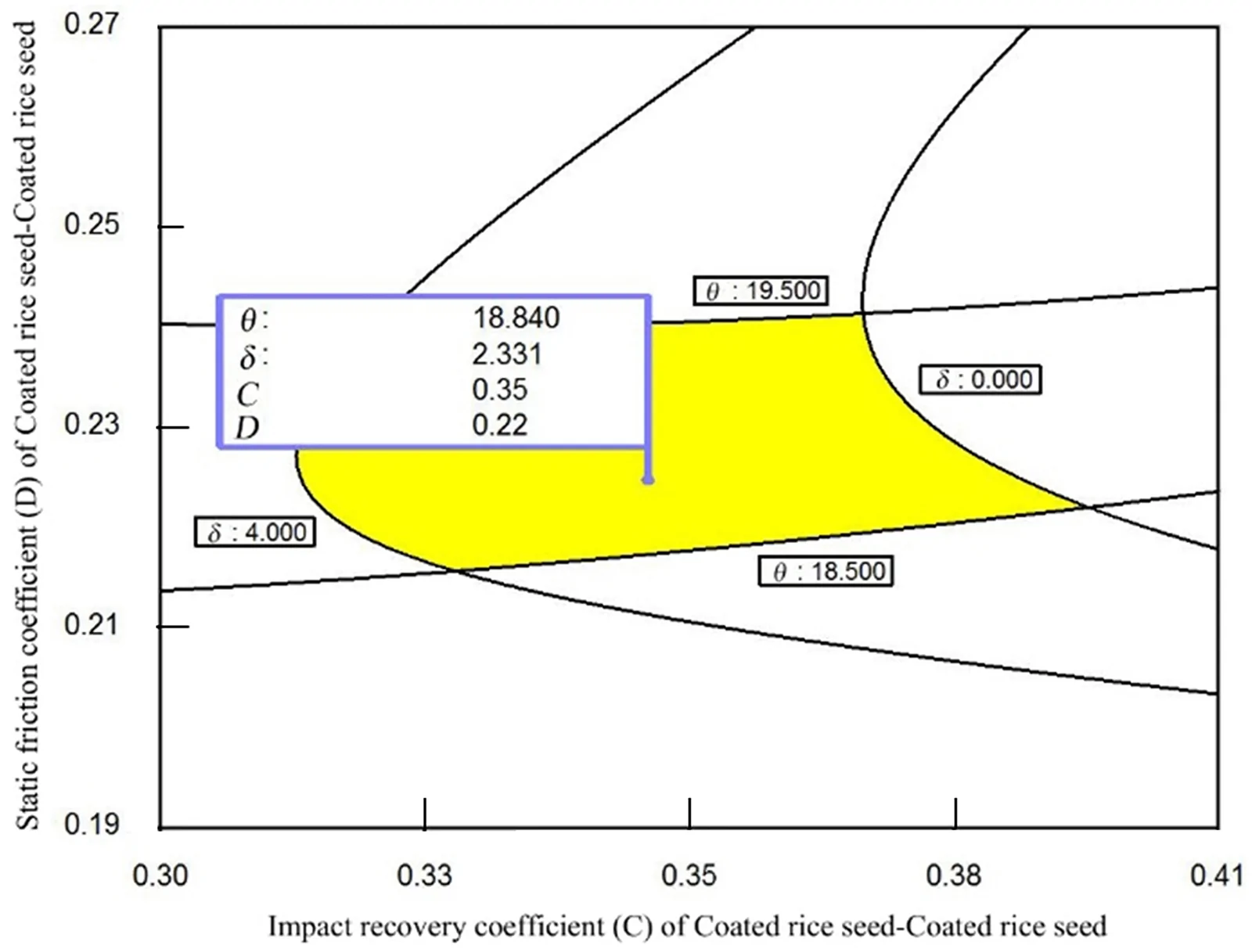
Optimization results of central combination parameters.

**Fig 13:**
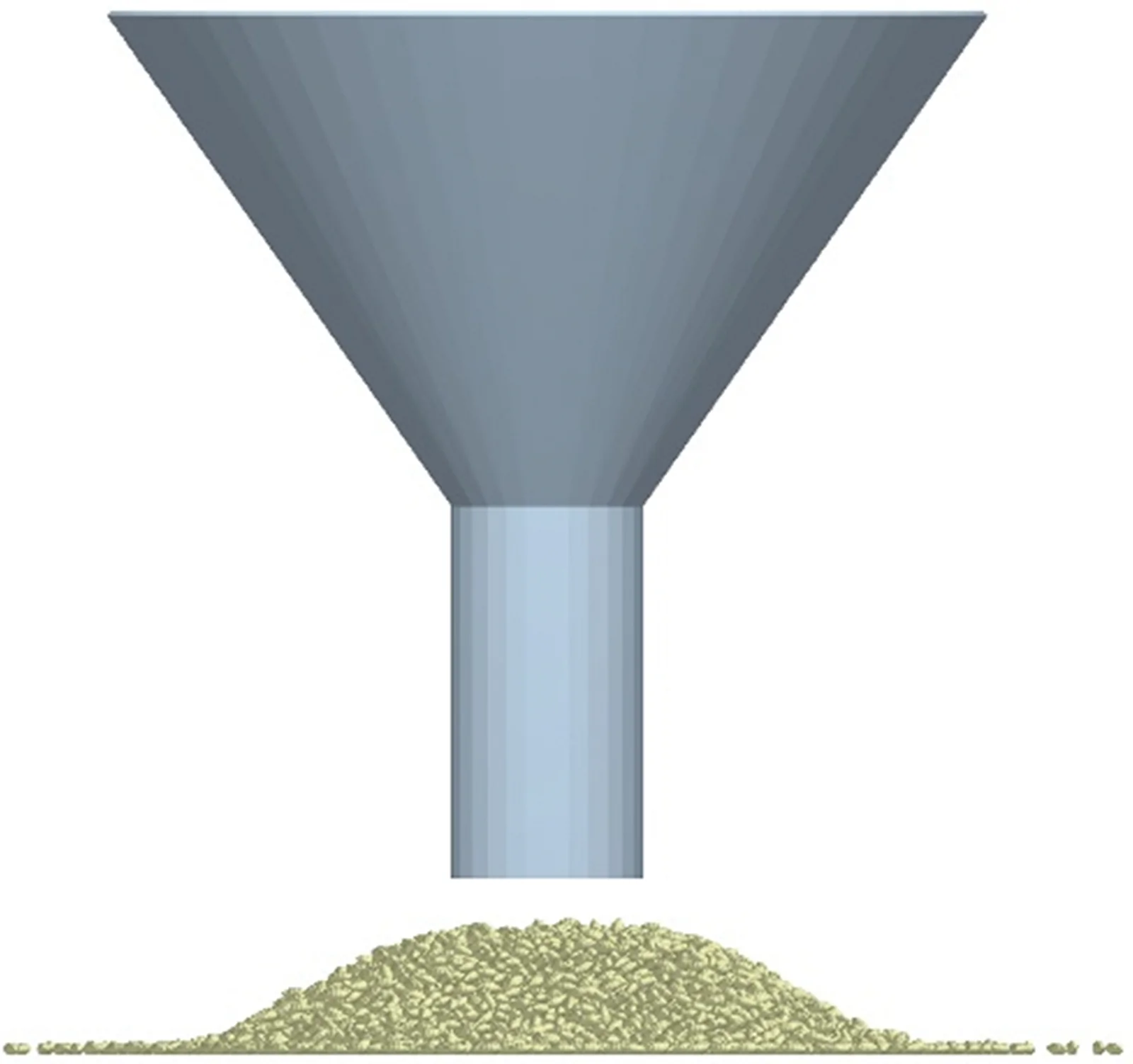
Simulated repose angle test of coated rice seeds.

### 3.4. Validation tests

#### 3.4.1. Static verification test.

In this study, static validation tests were conducted using the cylinder lifting method. During the test, coated rice seeds flowed out from a slowly and vertically lifted cylinder, forming a conical pile on a horizontal surface, and the static repose angle was measured. The measurement apparatus mainly consisted of a stainless steel cylinder and a stainless steel base plate, as shown in Fig 14. The cylinder had a diameter of 50 mm and a height of 350 mm, and was filled with 8,000 coated rice seeds. The cylinder was then lifted slowly, allowing the seeds to flow out from the bottom under gravity and accumulate on the base plate, forming a seed pile. The measured and simulated repose angles were compared through bench tests, with each test repeated three times and the average value taken. The results showed that the measured repose angle was 28.37°, and the simulated value was 28.63°, yielding a relative error of only 0.93%. The small error indicates that the discrete element model and simulation parameters for coated rice seeds are accurate and suitable for use in related DEM simulation studies.

**Fig 14:**
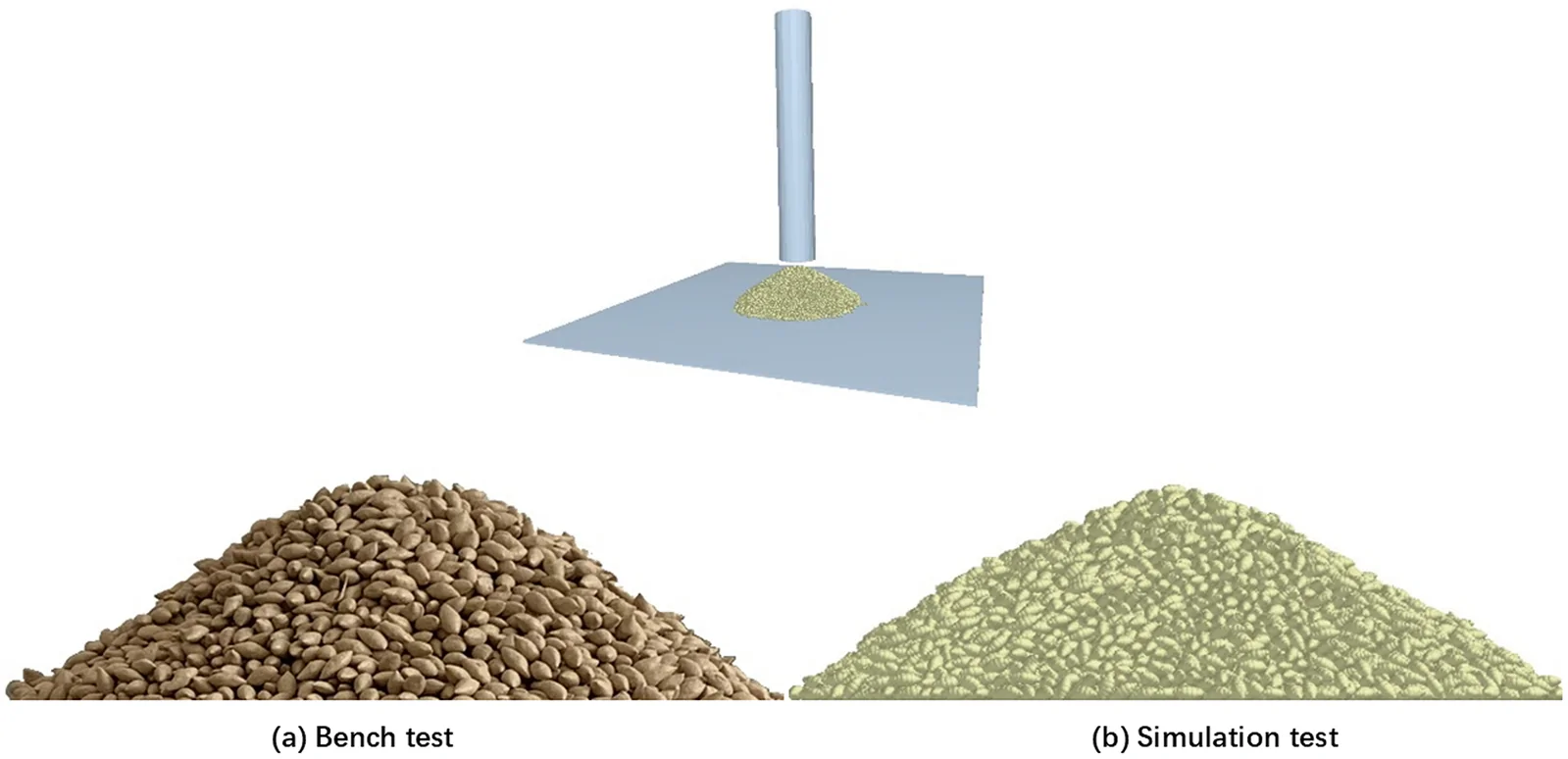
Static verification test.

#### 3.4.2. Dynamic verification test.

In this study, dynamic validation tests were conducted using the cylinder rotation method. To further assess the reliability of the discrete element model and simulation parameters for coated rice seeds, the seeds were allowed to move inside a rotating cylinder during the test, and the dynamic repose angle—defined as the angle between the inclined surface of the seed pile and the horizontal plane—was measured. The experimental setup mainly consisted of a stainless steel cylinder and a servo motor system, as shown in Fig 15. The cylinder had an inner diameter of 200 mm and a depth of 50 mm, and was filled with approximately 8,000 coated rice seeds. The outer side was sealed with a transparent acrylic plate to allow observation. As the cylinder rotated, the seed pile in the measurement region formed a stable inclined surface under the combined action of gravity and cylinder motion, and the angle between its generatrix and the horizontal plane was measured. Each test was repeated three times, and the average value was taken. A comparison between the measured and simulated values at different rotational speeds is presented in Table 10. The average relative errors were all below 5%, indicating that the calibrated model and parameters are reliable and suitable for use in subsequent discrete element simulation studies of coated rice seeds.

**Table 10:** The dynamic verification test data.

Speed(RPM)	Measured value(°)	Simulative value(°)	Error
4	39.93	38.48	3.63%
8	41.31	41.97	1.60%
12	43.43	43.95	1.20%
16	46.12	47.63	3.27%
20	48.02	48.83	1.69%

**Fig 15:**
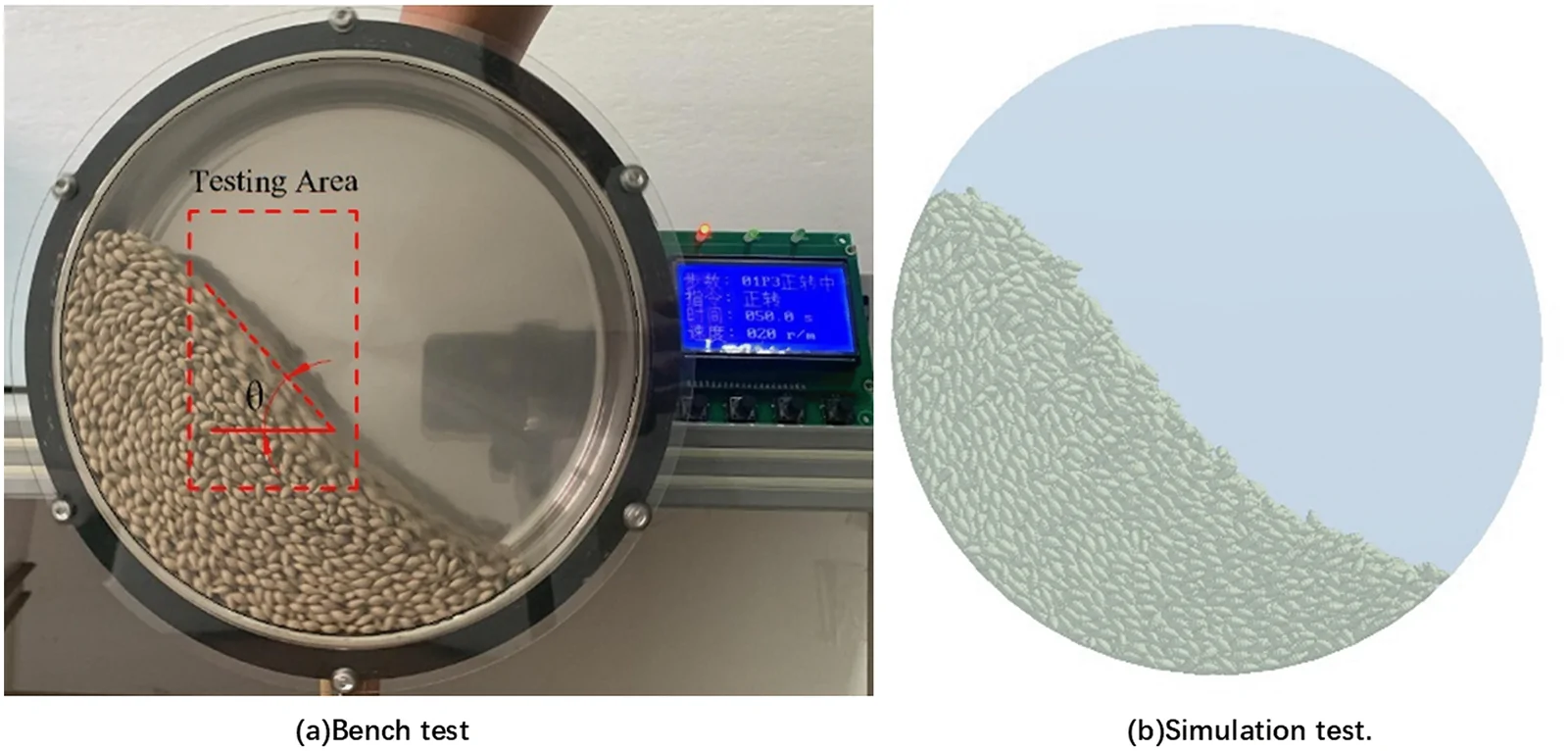
Dynamic verification test.

#### 3.4.3. Verification test of total seeding rate for UAV seeding device.

To further verify the applicability of the established discrete element model and simulation parameters of coated rice seeds in practical operations, this study carried out verification experiments on the total seeding rate using a UAV seeding device. The simulation boundary settings, test dimensions and operating conditions were strictly consistent with the actual outdoor test environment to ensure the validity of model verification. The experiments were performed with the centrifugal disc seeding device of a T40 agricultural unmanned aerial vehicle, where the opening degrees of the seeding bin gate were set to 100% and 50%, respectively. The total seeding rate of coated rice seeds was measured by comparing the actual seeding test with the simulation test, as shown in Fig 16. The collecting device has dimensions of 505 mm × 350 mm × 235 mm in length, width and height, which can prevent coated rice seeds from jumping out. All collecting devices are placed side by side. The length and width of the bin size for data sampling in the simulation are consistent with those of the actual collecting device. All tests were carried out under windless conditions. Each test was repeated three times, and the average value was calculated. The experimental data are presented in Table 11. The results demonstrate that the relative errors between the simulated and measured values under all working conditions are less than 3%, indicating that the discrete element model can be reliably applied to the simulation study on the seeding process of coated rice seeds.

**Table 11:** Experimental data of total seeding rate.

**Seeding gate opening**		100%	50%
Actual total seeding rate(kg/min)	Test 1	56.64	10.99
	Test 2	57.24	11.26
	Test 3	57.23	11.2
	Mean	57.04	11.15
Simulated total seeding rate(kg/min)	Test 1	55.35	10.63
	Test 2	54.96	12.05
	Test 3	56.48	11.64
	Mean	55.60	11.44
Relative Error		2.52%	2.60%

**Fig 16:**
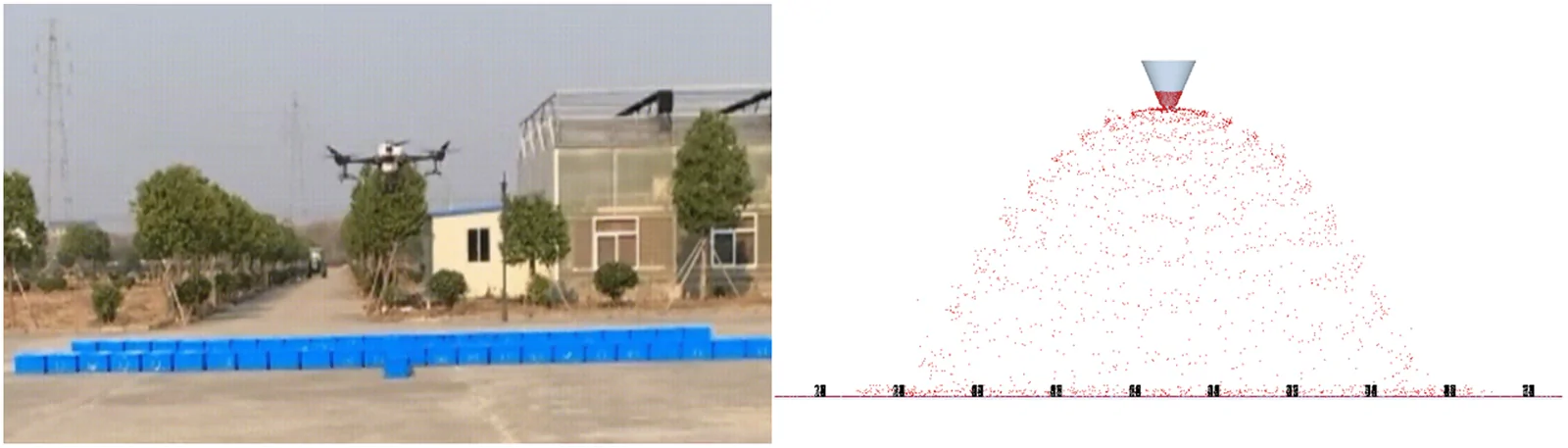
Verification test of total seeding rate for UAV seeding device.

### 3.5. Discussion

This section compares the present work with existing literature, and summarizes the innovations, limitations and future prospects. Most previous studies relied on empirical parameter selection and adopted only a single optimization method, lacking systematic multi-stage screening and optimization. Model verification was also limited to simple static tests, without combined dynamic and UAV field validation. This study innovatively adopted a three-stage progressive strategy: Plackett–Burman test for key parameter screening, steepest ascent test for optimal interval determination, and quadratic rotation orthogonal test for precise optimization and regression modeling. Meanwhile, static, dynamic and UAV seeding rate tests were combined to verify model accuracy, which improves the rationality of parameter calibration and the practical applicability of the simulation model. The main limitations are that only typical parameters and conventional working conditions were considered, and complex environmental factors were not included. The model applicability beyond the given parameter range still needs further verification. Subsequent research will incorporate more influencing factors and field conditions to enhance the generalization performance of the model for UAV precision seeding simulation.

## 4. Conclusion

1. The ANOVA test results indicated that among the simulation parameters, the CRS-CRS coefficient of restitution (C) had no significant effect on the repose angle (*θ*). In contrast, the CRS-CRS coefficient of static friction (D), the CRS-CRS coefficient of rolling friction (E), and the CRS-stainless steel coefficient of static friction (G) for coated rice seed–stainless steel contact all exhibited highly significant effects on the repose angle (*θ*). Specifically, the simulated repose angle (*θ*) increased with increasing values of D, E, and G. Regarding the relative error of the repose angle (*δ*), the CRS-CRS coefficient of restitution (C) and the CRS-CRS coefficient of rolling friction (E) showed no significant influence. However, the CRS-CRS coefficient of rolling friction (E) had a highly significant effect on *δ*, while the CRS-stainless steel coefficient of static friction (G) had a significant effect. The simulated repose angle error (*δ*) first decreased and then increased with increasing values of D and G.
2. The results of the quadratic rotation orthogonal combination test indicated that the established second-order regression model for the repose angle exhibited good reliability and accuracy. Furthermore, the optimal combination of simulation parameters for coated rice seeds was obtained as follows: the CRS-CRS coefficient of restitution (C) was 0.35; the CRS-CRS coefficient of static friction (D) was 0.22; the CRS-CRS coefficient of rolling friction (E) was 0.09; the CRS-stainless steel coefficient of restitution (F) was 0.4; the CRS-stainless steel coefficient of static friction (G) was 0.51; and the CRS-stainless steel coefficient of rolling friction (H) was 0.125.
3. Validation tests of the static repose angles of coated rice seeds under the optimal parameter combination demonstrated that the average relative error between the simulated and measured values was 0.93%, indicating no significant difference. Furthermore, the morphology of the seed pile in the simulation closely replicated that observed in the physical test. For the dynamic repose angle, the average relative errors between the measured and simulated values were all less than 5%, confirming a high level of agreement. Additionally, verification tests using a UAV seeding device showed that the relative errors in seeding rate between the simulated and measured values under all operating conditions were below 3%. These results collectively validate the reliability and accuracy of the established discrete element model and its parameters for coated rice seeds. The findings of this study provide essential data and methodological references for future discrete element simulations involving the mechanical collecting, screening, and mixing of coated rice seeds.

## Supporting information

S1 Data Raw experimental and simulation data. This file contains raw measurement data of coated rice seed physical properties, discrete element simulation calibration results, and statistical data of the UAV seeding test used in this study. These raw data underpin the results and conclusions reported in the manuscript.
(XLSX)
